# Retrotransposon-Derived Promoter of Mammalian *Aebp2*

**DOI:** 10.1371/journal.pone.0126966

**Published:** 2015-04-27

**Authors:** Hana Kim, Arundhati Bakshi, Joomyeong Kim

**Affiliations:** Department of Biological Sciences, Louisiana State University, Baton Rouge, Louisiana, United States of America; Ludwig-Maximilians-Universität München, GERMANY

## Abstract

Variable DNA methylation in promoter regions has been implicated in altering transcriptional regulation. The current study analyzed the evolutionary origin and DNA methylation pattern of one of the promoters of *Aebp2*. According to the results, the first promoter of *Aebp2* has been derived from retrotransposons independently in the primate and rodent lineages. DNA methylation analyses revealed that this promoter is unmethylated in sperm, methylated in mature oocytes, and partially methylated at embryonic day 10.5 (78.3%) and 14.5 (58.3%). This promoter also shows variable levels of DNA methylation among adult organs, ranging from the highest in spleen (~80%) to the lowest in tail (~50%). The results from the F1 hybrid of interspecific crossing further indicated that both alleles are equally methylated without any allele bias, also supported by its biallelic expression. Therefore, the partial methylation observed among somatic tissues is an outcome of the genome-wide resetting of DNA methylation during the implantation stage, but not of the inherited allelic methylation pattern preset during gametogenesis. Taken together, mammalian *Aebp2* has adopted retrotransposons as its promoter, which displays partial DNA methylation pattern of allelic- or non-allelic origin during the different stages of development.

## Introduction

*Aebp2* (Adipocyte Enhancer Binding Protein 2) is an evolutionarily well-conserved zinc finger gene that is known to interact with Polycomb Repressive Complex 2 (PRC2) [1–4]. As a DNA-binding protein of the PRC2, *Aebp2* is predicted to recruit the histone-modifying complex to a large number of developmental genes for their repression *via* H3K27me3 (trimethyation at lysine 27 of histone H3) [4–6]. The histone-modifying activity of PRC2 is the most obvious in stem cells and during early embryogenesis, thus the components of the PRC2, including *Aebp2*, are highly expressed in these cell types [3],[6]. Mammalian *Aebp2* encodes two protein isoforms with different expression patterns: embryonic (32 kDa) and somatic (52 kDa) forms [5]. In contrast, the other vertebrates have only one form of AEBP2, which is similar to the embryonic form of mammalian AEBP2 [5]. Thus, the embryonic form is likely an ancestral form whereas the somatic form represents a novel form unique to the mammalian lineage. In the mouse, the expression of the two protein isoforms is controlled through three alternative promoters with different expression profiles: the first and third (P1 and P3) with early stage-specific expression versus the second (P2) with ubiquitous expression [5],[7]. Interestingly, the genomic region surrounding the first promoter of mouse *Aebp2* overlaps with murine retrotransposons, whereas those for the second and third promoters overlap with CpG islands, a typical sequence structure of mammalian promoters [7]. Although intriguing, it is currently unknown why and how this retrotransposon-derived promoter has been selected for murine *Aebp2*.

The identification of the retrotransposon-derived promoter for *Aebp2* is very unusual and unexpected since the majority of retrotransposons are repressed through DNA methylation in mammalian genomes [8–10]. Although rare, there are several cases where retrotransposons escape from DNA methylation, and become temporary promoters driving the ectopic transcription of nearby endogenous genes, such as *A* ^*vy*^ (Agouti variable yellow) and *Axin* ^*fu*^ (Axin-fused) loci of the mouse genome [11],[12]. Interestingly, these ectopic promoters are known to display unusual DNA methylation patterns, which prompted us to test whether the retrotransposon-derived promoter of mouse *Aebp2* also has an unusual pattern of DNA methylation. Thus, the DNA methylation profiles of this promoter were analyzed in detail in the current study. According to the results, this retrotransposon-derived promoter is unmethylated in sperm but methylated in oocytes. However, this allele-specific methylation pattern is reset during the implantation stage. This promoter is also partially methylated but with variable levels among adult organs. Overall, this promoter displays two different types of DNA methylation patterns of allelic and non-allelic origins during the different phases of development. More detailed results are presented below.

## Results

### Retrotransposon-derived P1 promoter of mouse and human Aebp2

**Fig 1:**
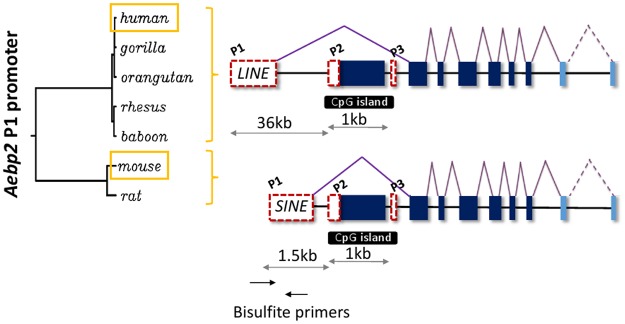
Retrotransposon-derived P1 promoter of human and mouse *Aebp2*. The phylogeny tree shown left was generated using the genomic sequences corresponding to the P1 promoter regions of five primate and two rodent species. The exon structures of human and mouse *Aebp2* are shown right: the dotted red boxes indicate three promoters whereas the closed boxes indicate exons.

According to previous studies, mouse *Aebp2* is controlled through three alternative promoters (Fig 1). The genomic regions surrounding the second (P2) and third (P3) promoters display CpG-rich sequence structures, and also high levels of sequence conservation among different mammals. In contrast, the 5’ end of the first promoter (P1) is occupied by two different types of murine SINEs (Short INterspersed DNA Elements), B1 and B2. Sequence comparison analyses further revealed that these two types of SINEs are also localized upstream of rat *Aebp2* with 84% sequence identity to the mouse region (S1 Fig). However, these two SINEs were not found in the *Aebp2* locus of the other mammals, and thus the P1 promoter is likely specific to the murine lineage without any conservation to the other mammalian lineage (S1 Fig). Since both rat and mouse have the P1 promoter that is made of these two elements, the formation of this promoter is estimated to be at least 15–20 million years ago, before the split of these two rodents [13]. The majority of retrotransposons that were amplified before the split of rat and mouse are no longer recognizable at the current evolutionary time due to the fast neutral mutation rate in the rodent genomes [14]. Nevertheless, the P1 promoter with these two elements still maintains 84% sequence identity between the two species, suggesting that these two elements have been functionally selected as a promoter for *Aebp2*. This is also supported by high levels of CpG content found within these two elements, 12 sites within one 200-bp region (S1 Fig), which are known to decay very rapidly into CpA or TpG due to the DNA methylation followed by deamination [15].

Human *AEBP2* also has three alternative promoters based on the exon structure predicted through mRNA and EST sequences (Fig 1 and S4 Fig). The two evolutionarily conserved promoters, P2 and P3, are localized similarly as seen in mouse *Aebp2*. However, the P1 promoter of human *AEBP2* is located 36-kb upstream of P2 and P3, yet the 600-bp genomic region surrounding this promoter overlaps again with another retrotransposon, a member of the L1PBa family, that was amplified around 50–60 million years ago in the primate genome [16]. The human *AEBP2* P1 shares 78% sequence homology with the 5’ UTR of the L1PBa transcript, and the 5’ end of the truncated L1 is inserted in the reverse orientation to human *AEBP2* transcript. According to the results from database searches, this element is found upstream of the *AEBP2* locus of the great apes as well as the old world monkeys, including baboon, rhesus and gibbon, with the high levels of sequence identity to the human region, ranging from 93 to 98%. As seen in the mouse, the human P1 promoter also maintains high levels of CpG content, 11 CpG site within a 200-bp region. The results from epigenome projects also indicated that this region is protected from DNA methylation during spermatogenesis [17], further supporting an idea that this region has been selected as a promoter for human *AEBP2*. In summary, the P1 promoter of *Aebp2* has been independently derived from two different types of retrotransposons in the two lineage of mammals during evolution, L1 for primates and SINEs for rodents.

### DNA methylation of the P1 promoter during gametogenesis and embryogenesis

**Fig 2:**
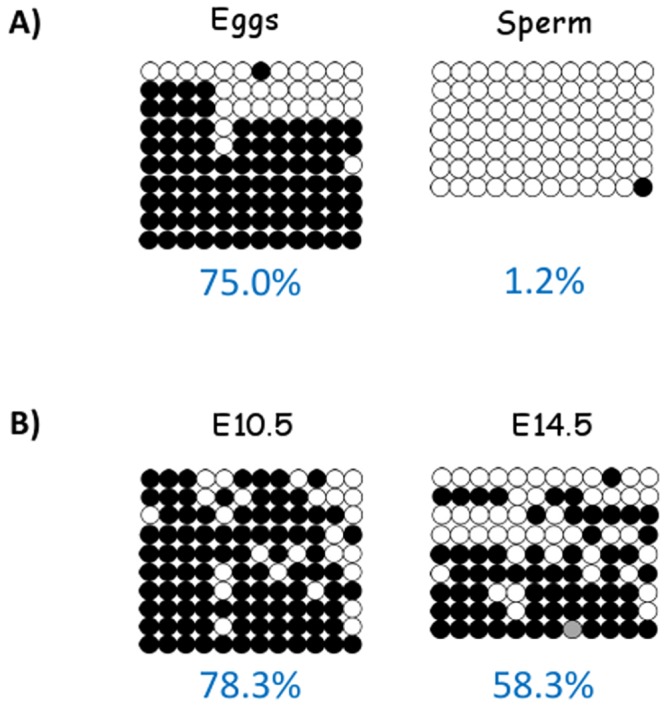
DNA methylation of the P1 promoter during gametogenesis and embryogenesis. The DNA methylation levels of the P1 promoter were analyzed using the DNA isolated from eggs and sperm (A) and E10.5 and E14.5 embryos (B). White and black circles represent unmethylated and methylated CpG dinucleotides, respectively. The overall methylation level of each sample is shown at the bottom.

The P1 promoter of mouse *Aebp2* was further analyzed in terms of DNA methylation given its intrinsic nature, attracting DNA methylation, as an inserted retrotransposon. DNA was isolated from various tissue samples representing the different stages of development. The isolated DNA was treated with the bisulfite conversion protocol, and subsequently used for PCR amplification [18]. The amplified PCR products were analyzed with sequencing (Fig 2). The P1 promoter was completely unmethylated in sperm, indicating that this region is protected from DNA methylation during spermatogenesis, which is consistent with the results from the P1 promoter of human. In contrast, the P1 promoter shows 75% DNA methylation level in mature oocytes, which is quite opposite to the result from sperm. The methylation pattern from mature oocytes appears to be somewhat sequencing read-specific: the majority of sequence reads are 100% methylation whereas a few reads are completely unmethylated. This implicates that the levels of DNA methylation are likely variable among individual oocytes. DNA isolated from two embryonic stages, E10.5 and E14.5, were also analyzed in a similar manner. The P1 promoter displays 78.3% methylation level at E10.5 and 58.3% methylation level at E14.5. The methylation patterns at both stages are more or less mosaic, which is different from the sequencing read-specific pattern that was seen in the mature oocytes (Fig 2). Overall, this series of DNA methylation analyses concluded that the P1 promoter of mouse *Aebp2* is unmethylated in sperm, but methylated in oocytes, and partially methylated at E10.5 and E14.5 stages.

### Non-allelic partial DNA methylation pattern of the P1 promoter

**Fig 3:**
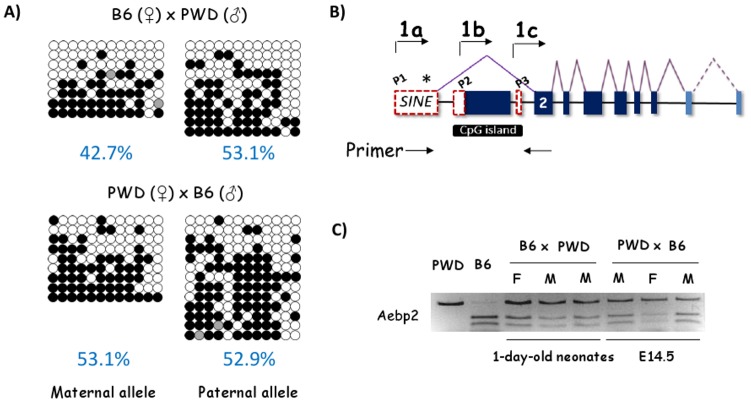
Non-allelic, partial DNA methylation pattern of the P1 promoter. (A) The DNA methylation levels of the P1 promoter were analyzed using one set of F1 hybrid offspring derived from the reciprocal crosses between C57BL/6J and PWD/PhJ. Individual sequencing reads were separated based on the parental origin. White and black circles represent unmethylated and methylated CpG dinucleotides, respectively. Gray circles indicate the CpG dinucleotides with unknown methylated status. (B) RT-PCR scheme for imprinting test. Two primers targeting the P1-driven transcript were shown as arrows underneath the exon structure of mouse *Aebp2*. The asterisk (*) inside Exon1a indicates one sequence polymorphism between C57BL/6J and PWD/PhJ, which was used for differentiating two alleles by *HpyCH*4III restriction enzyme digestion of the RT-PCR product. (C) Biallelic expression of the P1-driven transcript. Two sets of RT-PCR products derived from E14.5 embryos (lanes six-eight) and one-day-old pups (lanes three-five) were digested with the restriction enzyme to test their allelic origin. The digestion patterns were also compared with those of two parental strains (lanes one and two).

Since the P1 promoter suggests an allele-specific methylation pattern, based on the DNA methylation levels in the germ cells, we performed another series of DNA methylation analyses using the DNA isolated from the F1 hybrid of interspecific crossing between C57BL/6J and PWD/PhJ. DNA was isolated from the tails of one-day-old pups that had been derived from the F1 hybrids of two reciprocal crosses (Fig 3A). The isolated DNA were treated similarly as described earlier. Two sequence polymorphisms found between the two subspecies of mouse were used for differentiating two parental alleles. According to the results, the P1 promoter showed 42.7 and 53.1% methylation on maternal and paternal alleles, respectively, of the F1 hybrid derived from the crossing between female C57BL/6J and male PWD/PhJ. The observed methylation difference between the two alleles, 42.7 and 53.1%, was not statistically significant (p = 0.11) according to Fisher’s exact test. This is also the case for the second F1 hybrid derived from the reciprocal cross between female PWD/PhJ and male C57BL/6J, showing 53.7 and 52.9% methylation on the maternal and paternal alleles, respectively. The methylation patterns observed from these two sets were overall mosaic. More importantly, the methylation levels of both alleles are similar, ranging from 42 to 53%, without any allele bias.

The observed DNA methylation pattern of the P1 promoter was further investigated with a series of imprinting tests using the total RNA isolated from two sets of crosses (Fig 3B). The first set of RNA were derived from the 1-day-old F1 hybrids from the crossing between the female C57BL/6J and male PWD/PhJ (lanes three through five), whereas the second set from the 14.5-day-old F1 embryos from the crossing between female PWD/PhJ and male C57BL/6J (lanes six and eight). These RNA were reverse transcribed and subsequently amplified with PCR. The differentiation of two alleles was achieved through a restriction enzyme digestion-based assay utilizing one sequence polymorphism localized within Exon1a. According to the results, the transcript driven by the P1 promoter was derived from both alleles at similar levels. This observation was true for both reciprocal crosses and also for both developmental stages. Overall, the bi-allelic expression of the P1-driven transcript is consistent with the DNA methylation pattern of the P1 promoter, showing the partial methylation pattern with no allele bias.

### Variable levels of DNA methylation of the P1 promoter among adult organs

**Fig 4:**
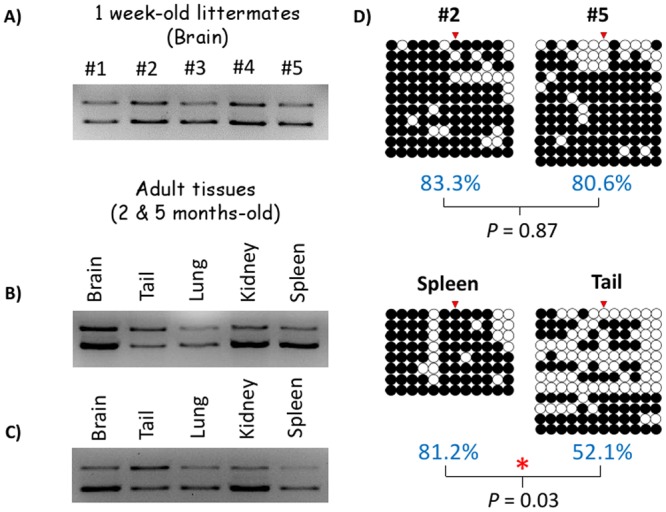
Variable levels of DNA methylation of the P1 promoter among neonates and adult organs. The DNA methylation levels of the P1 promoter were analyzed using the first set of DNA derived from one-week-old littermates (A), the second and third sets from the various organs of two- (B) and five-month-old individual mice of C57BL/6J (C). The PCR products amplified from the bisulfite-converted DNA were digested with *Taq*I enzyme to survey the methylation levels. The upper and lower bands indicate the undigested and digested, thus unmethylated and methylated DNA fragments, respectively. Two samples (#2 and #5) from the first set (one-week-old littermates) and two additional samples (Spleen and Tail) from the third set (five-month-old mice) were further analyzed through cloning and sequencing (D). The methylation levels are presented along with the *P*-values from Mann-Whitney U test measuring statistical significances.

Since the P1 promoter shows partial methylation during early embryogenesis, the methylation patterns and levels were further analyzed using DNA isolated from various tissues of different ages (Fig 4). For this series of experiments, we prepared the three sets of DNA that were derived from five one-week-old littermates and several organs of two and five-month old mice of C57BL/6J background. These three sets of DNA were treated with the bisulfite conversion, amplified with PCR, and finally the PCR products were analyzed first with COBRA and later with individual sequencing [18],[19]. As shown in Fig 4A, the P1 promoter shows partial methylation among the five littermates of one-week-old age, with no significant difference between the five individual brains (Fig 4A and 4D). On the other hand, the two other promoters, P2 and P3, were not methylated at all (S2 Fig). Among the individual tissues of two- and five-month-old mice, tail tends to show less methylation levels than the remaining other organs (Fig 4B,4C, and 4D). Some of these results were further confirmed through individual sequencing as shown in Fig 4D. While both one-week brains and the five-month spleens showed approximately 80% methylation levels, five-month tail tissue was significantly hypomethylated, showing only 52.1% methylation (P = 0.03 compared to 5-month spleen from same individual, Mann-Whitney U Test). In conclusion, the P1 promoter shows partial and variable methylation patterns among adult organs, which agrees with the partial methylation pattern observed from the embryonic stages. Thus, the P1 promoter appears to maintain partial and variable methylation patterns throughout development.

## Discussion

In the current study, we analyzed the evolutionary origin and DNA methylation pattern of one of the promoters of *Aebp2*. According to the results, the first promoter has been independently derived from retrotransposons in the primate and rodent lineage. This promoter is unmethylated in sperm and methylated in mature oocytes. This allele-specific methylation pattern is, however, reset during the implantation stage. This promoter is also partially methylated but with variable levels among adult organs. Overall, the mammalian *Aebp2* locus has adopted an inserted retrotransposon as its promoter along with its unusual DNA methylation patterns during the different stages of development.

**Fig 5:**
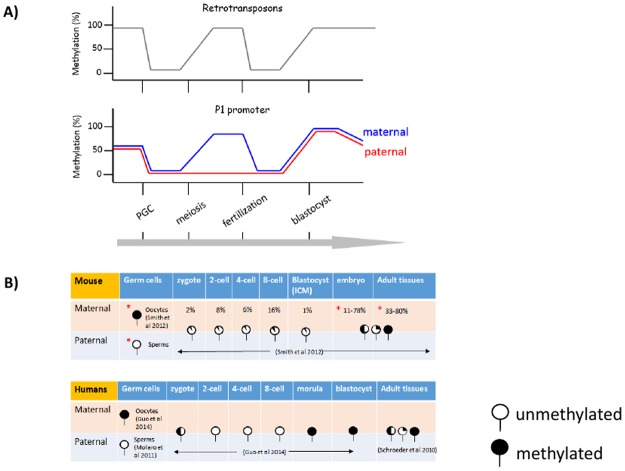
Summary of DNA methylation patterns of the P1 promoter during development. (*upper*) Retrotransposons as part of the mammalian genomes go through two rounds of DNA methylation setting as indicated by the fluctuating methylation levels (Y axis) during development (X axis). (*lower*) The retrotransposon-derived P1 promoter also goes through two rounds of the resetting process, but with two major modifications. First, the paternal allele (red line) does not regain DNA methylation during spermatogenesis. Second, the P1 promoter starts losing its DNA methylation levels at different rates between individual tissues after the implantation, resulting in varying levels of DNA methylation among adult organs (A). A table summarizing the overall changes of DNA methylation in Aebp2 P1 promoter in mouse [27] and human [17], [28], [29] during development (B). Developmental stages examined by COBRA analysis and bisulfite sequencing in this study are marked with red asterisks.

The methylation patterns of the P1 promoter during development can be summarized and compared with those of the other retrotransposons (Fig 5). Mammalian genomes go through two rounds of the resetting of DNA methylation: the first round during the transition period from the primordial germ cell (PGC) to germ cells and the second round during the early embryogenesis from fertilization to implantation stages [20],[21]. During the first resetting stage, the P1 promoter and the rest of retrotransposons lose similarly somatic cell-driven DNA methylation in the PGC of both genders. Later, the P1 promoter and the retrotransposons both regain DNA methylation during oogenesis, although the levels and patterns of DNA methylation on the P1 promoter are somewhat different from those on the retrotransposons. For instance, the level is somewhat lower than expected, 80%. Yet, some of this lower-than-expected methylation levels is contributed by the clonal variability of DNA methylation observed among individual oocytes (Fig 2). As a result, two different types of mature oocytes are possible: one with 100% and the other with 0% methylation on the P1 promoter. During spermatogenesis, on the other hand, the P1 promoter does not regain DNA methylation, which is quite different from the complete methylation pattern observed in the majority of retrotransposons. After fertilization, both paternal and maternal genomes lose DNA methylation again, but by different modes: the paternal genome by Tet- and BER (Base Excision Repair)-mediated active DNA demethylation whereas the maternal genome by replication-mediated passive DNA demethylation [21]. Thus, the maternal allele of the P1 promoter is likely demethylated by the passive replication-mediated mechanism. In the case of the paternal allele, the P1 promoter is already unmethylated, thus transcriptionally competent. It is well known that the zygotic genome of the mouse is active as early as at the two-cell stage [22]. Thus, the P1-driven transcript of *Aebp2* is likely contributed more by the paternal than maternal alleles. During the implantation stage, the majority of retrotransposons regain DNA methylation, and this methylation status is maintained throughout the embryogenesis and somatic cell differentiation. This is also the case for the P1 promoter based on the results (Figs 2 and 3). However, the established DNA methylation on the P1 promoter appears to be lost at different rates among individual cell types. As a consequence, its DNA methylation levels are variable among individual tissues (Fig 4).

The DNA methylation patterns of the *Aebp2* P1 promoter during development can be summarized and compared with those of the other retrotransposons (Fig 5). The time points examined in this study are marked in red asterisks (Fig 5B), and other genome-wide data sets used for P1 site CpG methylation analysis are listed in S2 Table. The large scale genome data provides a brief snap shot of some of the DNA methylation patterns, however, the coverage for Aebp2 P1 is insufficient. In addition, much of the RRBS (Reduced Representation Bisulfite Sequencing) data published, are missing the oocyte DNA methylation on P1 [23] possibly due to problems in mapping the repeat element from P1 promoter. Thus further analysis of human P1 is necessary to draw concrete conclusions of the dynamic changes in the human P1 promoter. Overall, the DNA methylation patterns of the P1 promoter are quite different from those of the other retrotransposons at two specific stages. First, the P1 promoter is hypomethylated in the sperm. Second, the DNA methylation in somatic cells is partial and variable, but the functional implications for this partial and variable methylation are currently unknown.

In each of the primate and rodent lineage, a retrotransposon of independent origin has become a promoter for *Aebp2*, representing a seemingly very unusual coincidence during mammalian evolution. Although there have been similar cases where retrotransposons have become promoters for the adjacent endogenous genes [11],[12], it is still very rare that similar events have occurred independently at the orthologous locus of two different mammalian lineages. Thus, this may not be a simple coincidence. Rather, it could represent an evolutionary outcome stemming from some functional needs that are associated with the mammalian *Aebp2* locus. According to the recent studies, the somatic form driven by the P2 promoter has been shown to function as an activator, which is opposite to the known repressor function by the embryonic form [7]. The activator role played by the somatic form may compromise the repressive role by the embryonic form, if they coexist. To circumvent this problem, the *Aebp2* locus might have taken advantage of some features associated with retrotransposons. As described above, the retrotransposon-derived P1 promoter displays a very unusual DNA methylation profile. In particular, the P1 promoter is temporarily unmethylated during the two resetting stages of DNA methylation, yet these are the stages when the PRC2 along with the embryonic form of AEBP2 play a major role in repressing the majority of developmental genes [3],[6]. Thus, the unmethylated P1 promoter serves as a temporary blocker to repress the P2 promoter responsible for the expression of the somatic form. One possible mechanism would be that RNA Pol II complex initiated at the upstream P1 promoter functions to block the accessibility of the downstream P2 promoter to the other components required for the initiation step of Pol II transcription. In that regard, it is interesting to note that the somatic form is a newly invented form specific to the mammalian lineage [5], which is somewhat parallel to the mammalian-specific formation of the P1 promoter from retrotransposons. These two mammalian-specific events, the formation of the somatic form and the P1 promoter, are connected and thus have co-evolved for the mammalian-specific functionality of the *Aebp2* locus. One possible scenario would be that the *Aebp2* locus have used randomly inserted retrotransposons for the temporary control of its somatic form during early embryogenesis. If this is the case, a similar situation should have occurred in the other mammalian lineage, which will be interesting to pursue in the near future. Overall, the mammalian *Aebp2* locus has adopted an inserted retrotransposon as its promoter along with its unusual DNA methylation patterns.

## Materials and Methods

### Ethics statement

All the experiments related to mice were performed in accordance with National Institutes of Health guidelines for care and use of animals, and also approved by the Louisiana State University Institutional Animal Care and Use Committee (IACUC), protocol #12–095.

### DNA methylation analyses

DNA methylation levels of the promoters of mouse *Aebp2* were analyzed using genomic DNA isolated from two germ cells, sperm and oocyte, as well as the various tissues of the mice with different developmental stages. Detailed protocols for isolating sperm and oocyte were described previously [24]. Briefly, sperm was isolated from the epididymus of three-month-old mice using the ‘swim-up’ method [25]. Mature oocytes were isolated from three-month-old females after superovulation with PMSG and hCG treatment [26], [27]. The isolated DNA was treated with the bisulfite conversion reaction according to the manufacturer’s protocol (EZ DNA methylation kit, Zymo Research). The converted DNA was used as a template for the PCR reaction using specific primers that were designed for amplifying the promoters of *Aebp2*. Each PCR product was further analyzed using the following two approaches: 1) the restriction enzyme digestion-based COBRA [19] and 2) cloning and sequencing. For the COBRA analysis, PCR products were digested with various restriction enzymes. Each PCR product was also individually cloned into the pGEM T-Easy vector (Promega), and 10 to 20 clones were subsequently sequenced to survey its DNA methylation levels at each locus. The detailed information regarding oligonucleotide sequences, sequence polymorphisms, and COBRA is also available (S1 Table).

### Statistical analysis

DNA methylation data for the imprinting test was analyzed using the non-parametric Fisher’s exact test, since two discrete numerical variables were tested to determine whether there is a parental allele specific pattern of complete (100%) methylation and complete unmethylation (0% methylation). Therefore, a 2X2 contingency table was used to test for parental allele-specific methylation pattern (Fig 3). Aebp2 P1 methylation patterns in various tissues at different stages, however, were analyzed using Mann-Whitney U test, to ascertain whether significant differences existed between continuous variables since the methylation ranges varied continuously between the samples tested (Fig 4).

### Mouse breeding and imprinting test

Embryos with the two different stages, 10.5 and 14.5, were obtained through timed mating between male and female mice of the C57BL/6J strain. The harvested embryos were used for DNA isolation and methylation analyses. For imprinting test, male and female mice of the C57BL/6J strain were bred individually with female and male mice of the PWD/PhJ strain (Jackson Lab, Stock No. 004660). One-day-old F1 pups from these crosses were sacrificed and used for isolating DNA and RNA. The isolated DNA was treated with the bisulfite conversion protocol for DNA methylation analyses. To test the mono-allelic expression (imprinting) of *Aebp2*, the isolated RNA was reverse-transcribed using the SuperScript III First-Strand Synthesis System (Invitrogen). The resulting cDNA was used as a template for PCR designed to amplify the transcribed region of *Aebp2* spanning Exon1a through 2. The RT-based imprinting test was conducted in three individuals (littermates) from each reciprocal cross. One of these individuals from the reciprocal cross was selected for bisulfite sequencing-based imprinting test.

All the detailed information regarding the primer sets for RT-PCR are available in S1 Table.

## Supporting Information

S1 Fig Sequence alignment of the P1 promoters derived from two rodent and five primate species along with randomly chosen L1 sequences. The CpG dinucleotides preserved during evolution are marked yellow while the CpG sites decayed to TpG are marked with blue. The predicted transcriptional start site (TSS) and transcriptional direction is marked in blue arrow.
(TIF)

S2 Fig Methylation levels of P2 and P3. COBRA data showing lack of DNA methylation at P2 and P3 in brains of five one-week-old littermates and various tissues from a five-month old mouse of C57BL/6J strain (A). The unmethylated status of P2 and P3 were further confirmed by bisulfite sequencing in two additional tissues from adult mice (B). White and black circles represent unmethylated and methylated CpG dinucleotides, respectively. Gray circles indicate the CpG dinucleotides with unknown methylation status.
(TIF)

S3 Fig *Aebp2* P1 and P2 promoter assay. Three mammalian cell lines, NIH 3T3, Neuro2A and HEK293T cells were transfected with plasmids (2μg) containing Aebp2 P1 and P2 promoters along with a luciferase reporter gene. All samples were co-transfected with and independent β-Geo reporter construct to monitor transfection efficiency and normalize all luciferase assay values.
(TIF)

S4 Fig Summary of mouse and human *AEBP2* P1 ESTs. The ESTs were blasted against the *Aebp2* P1 promoter sequence. The scores for mouse *Aebp2* P1 ESTs are from NCBI, and the human *AEBP2* P1 ESTs are scored from Blast2 of Aebp2 human P1 sequence (used for multiple sequence alignment in S1 Fig) against other ESTs ([30], NIH-MGC EST Sequencing project).
(TIF)

S5 Fig Genome wide distribution of 5’ UTR of human L1PBa. Red triangles indicate regions homologous to L1PBa 5’UTR.
(TIF)

S6 Fig Snap-shots of DNA methylation profiles and ESTs in L1PBa 5’ UTRs. Four snap shots of UCSC genome browsers are from regions marked in S5 Fig, which include regions homologous to L1PBa 5’ UTRs. Hypomethylation of DNA in sperm are marked in blue [17]. Available ESTs are added in the UCSC genome browser tracks. Browsed regions in genome (Hg19): Human AEBP2 P1 chr12:19557074–19557526; (I) chr2:199837446–199837987, (II) chr5:105204915–105205447, (III) chr11:13572036–13572117.
(TIF)

S1 Table Sequence and position information of the oligonucleotides that were used for RT-PCR and bisulfite sequencing for the current study. (XLSX)

S2 Table Summary of RRBS data used for generating table in Fig 5B. (XLSX)
