# Supplementary figures and images for: Retrotransposon-Derived Promoter of Mammalian Aebp2
Source: PLoS One. 2015 Apr 27;10(4):e0126966. doi: 10.1371/journal.pone.0126966 (PMC4411029; doi:10.1371/journal.pone.0126966)

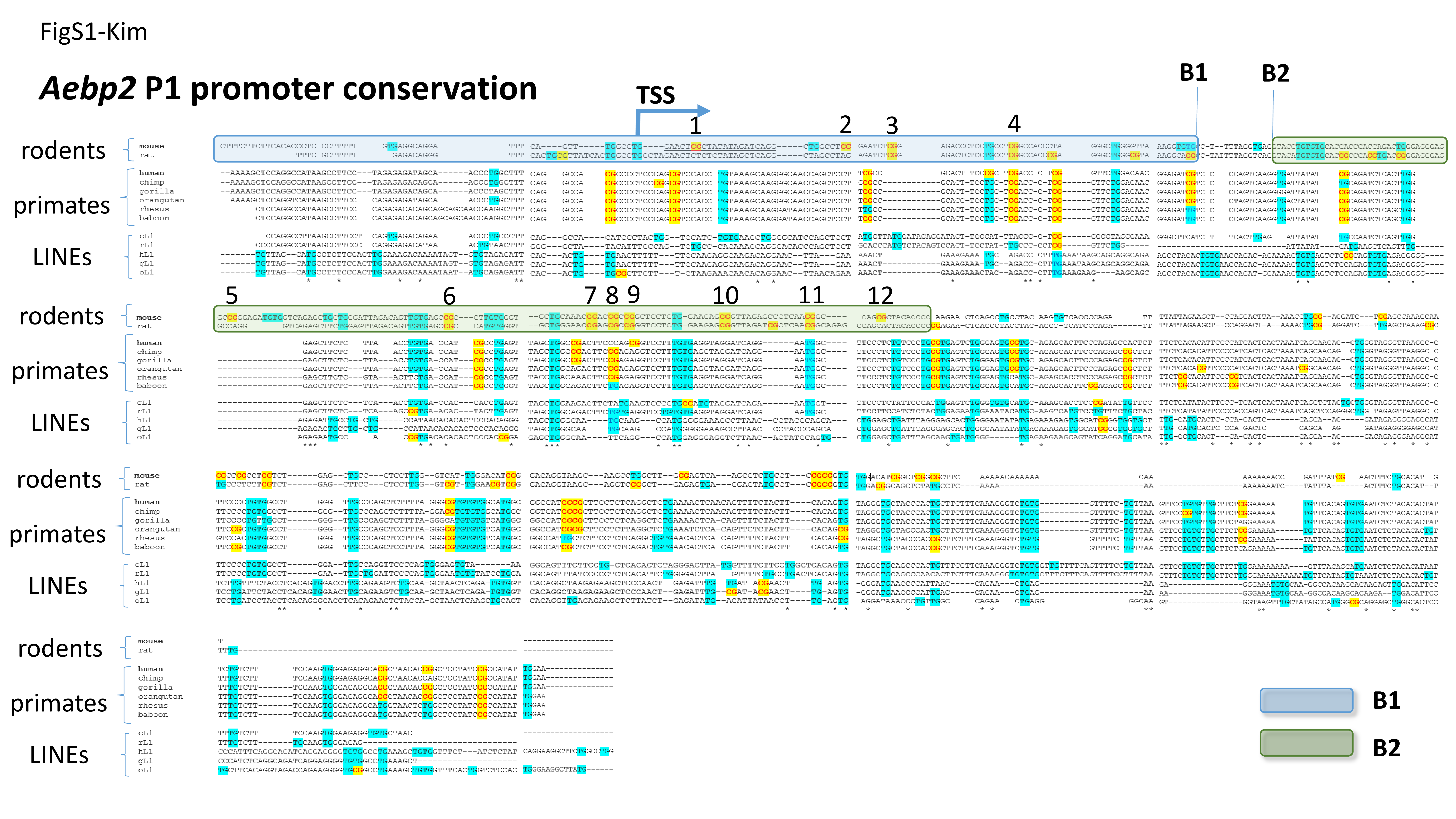

Supplement: S1 Fig — The CpG dinucleotides preserved during evolution are marked yellow while the CpG sites decayed to TpG are marked with blue. The predicted transcriptional start site (TSS) and transcriptional direction is marked in blue arrow. (TIF) [file pone.0126966.s001.tif]

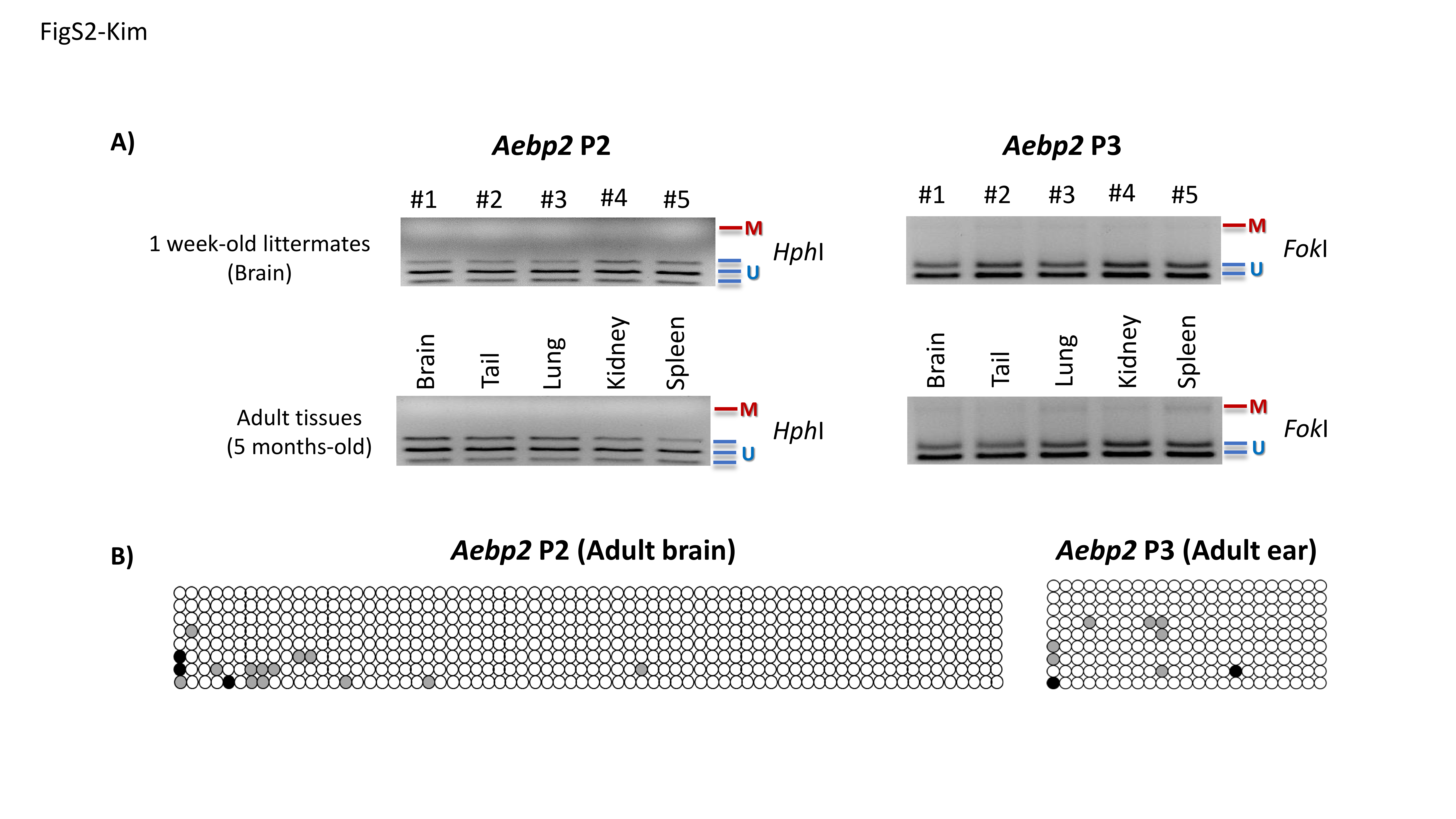

Supplement: S2 Fig — COBRA data showing lack of DNA methylation at P2 and P3 in brains of five one-week-old littermates and various tissues from a five-month old mouse of C57BL/6J strain (A). The unmethylated status of P2 and P3 were further confirmed by bisulfite sequencing in two additional tissues from adult mice (B). White and black circles represent unmethylated and methylated CpG dinucleotides, respectively. Gray circles indicate the CpG dinucleotides with unknown methylation status. (TIF) [file pone.0126966.s002.tif]

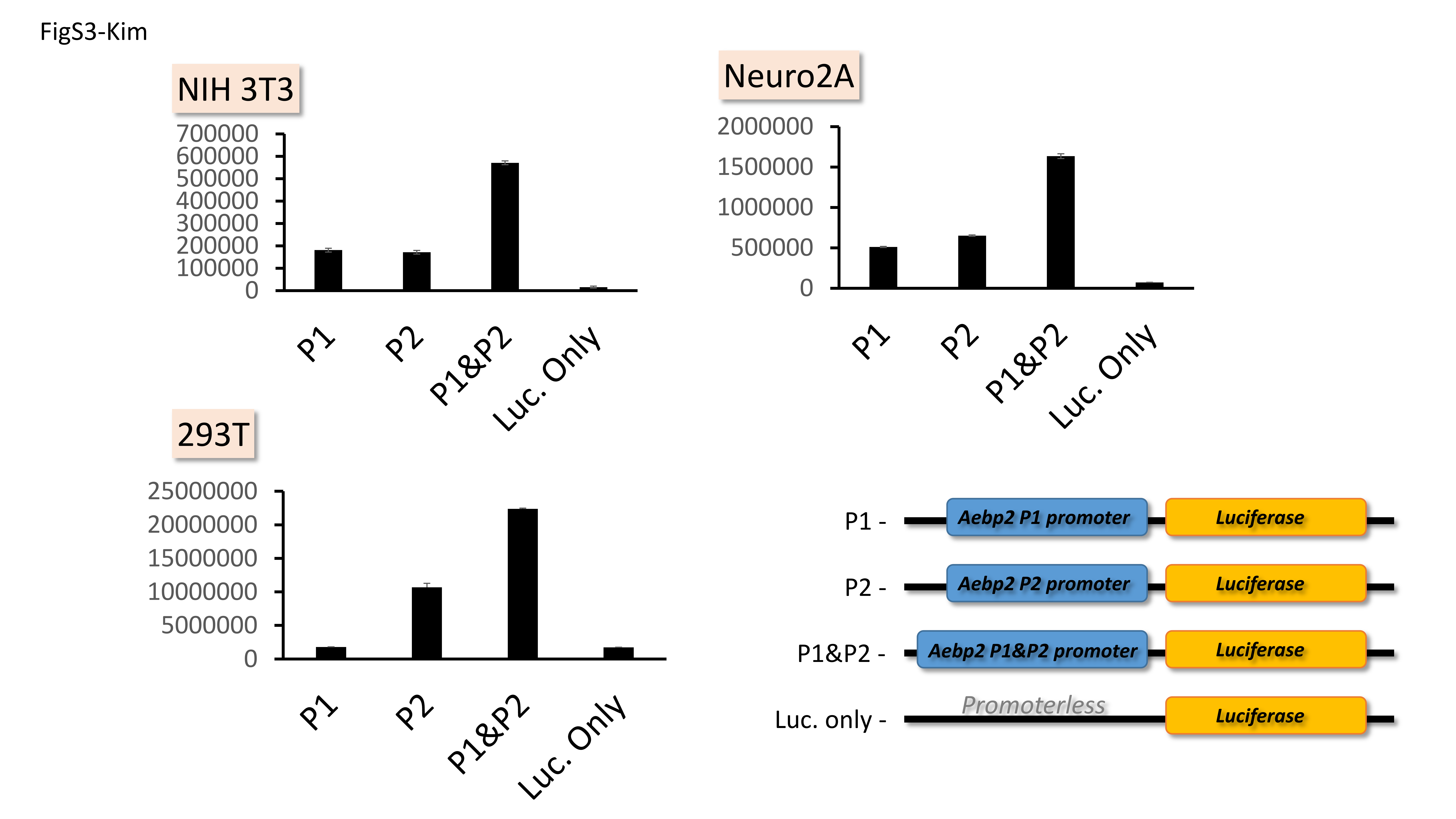

Supplement: S3 Fig — Three mammalian cell lines, NIH 3T3, Neuro2A and HEK293T cells were transfected with plasmids (2μg) containing Aebp2 P1 and P2 promoters along with a luciferase reporter gene. All samples were co-transfected with and independent β-Geo reporter construct to monitor transfection efficiency and normalize all luciferase assay values. (TIF) [file pone.0126966.s003.tif]

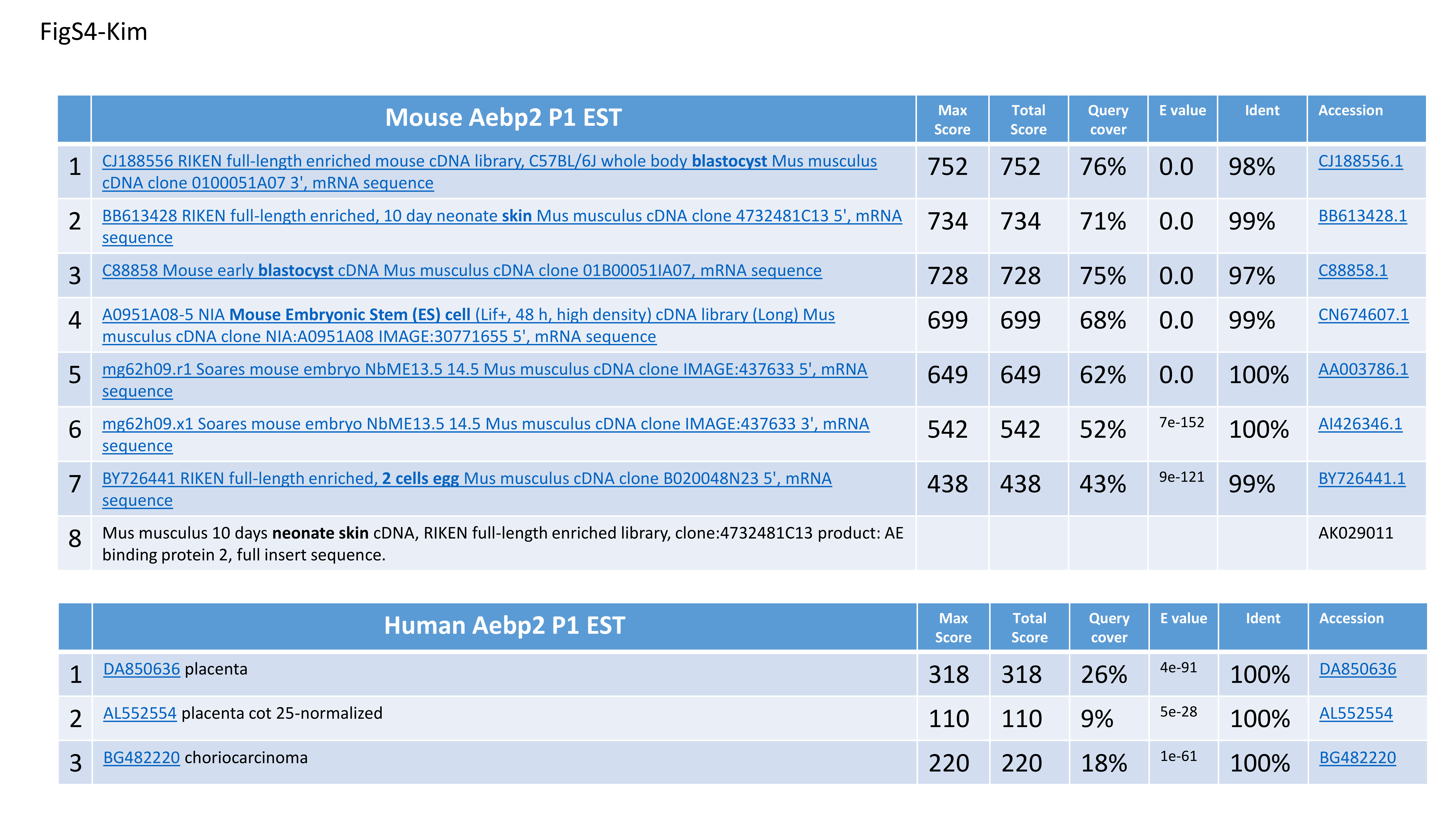

Supplement: S4 Fig — The ESTs were blasted against the Aebp2 P1 promoter sequence. The scores for mouse Aebp2 P1 ESTs are from NCBI, and the human AEBP2 P1 ESTs are scored from Blast2 of Aebp2 human P1 sequence (used for multiple sequence alignment in S1 Fig) against other ESTs ([30], NIH-MGC EST Sequencing project). (TIF) [file pone.0126966.s004.tif]

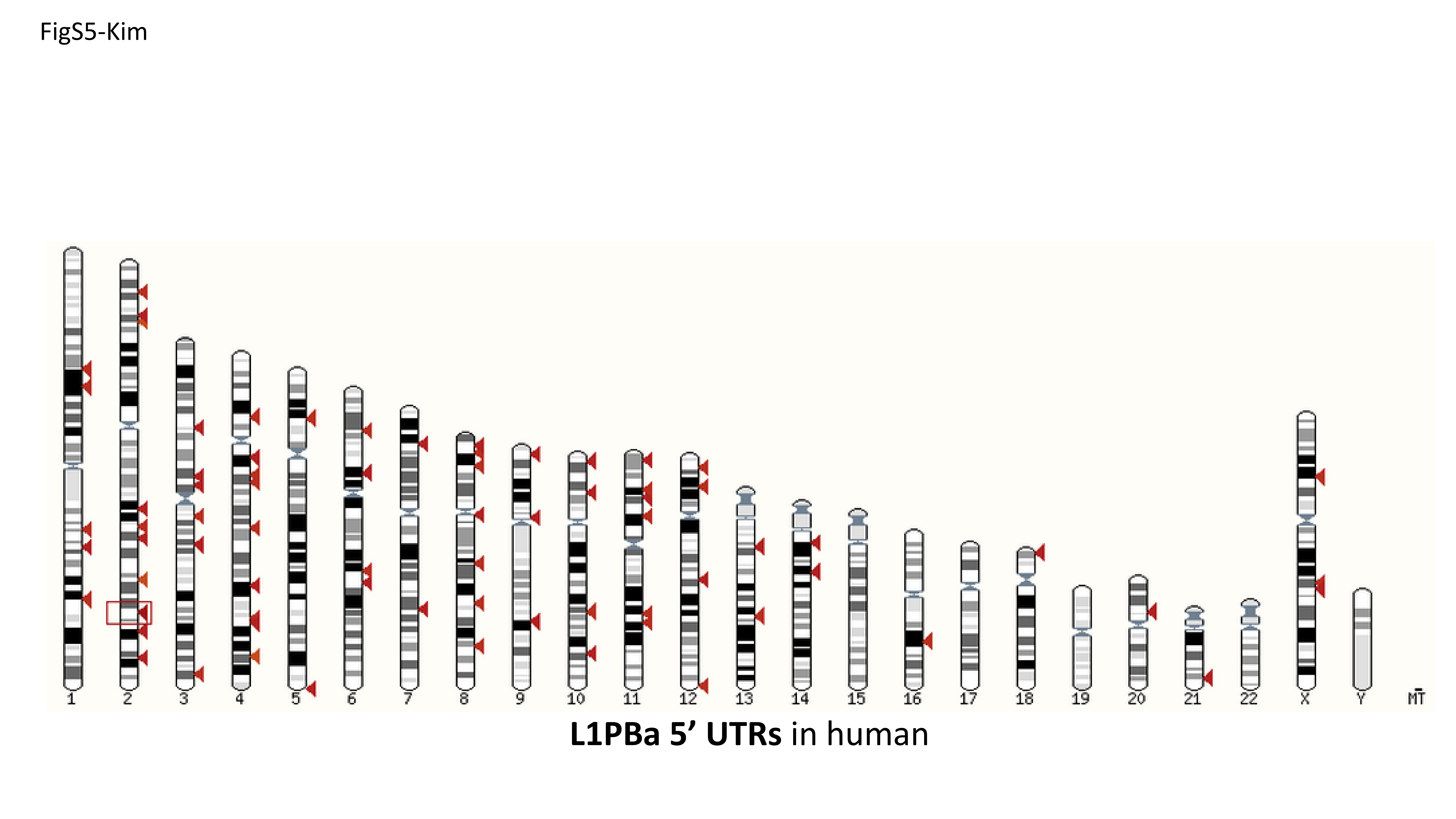

Supplement: S5 Fig — Red triangles indicate regions homologous to L1PBa 5’UTR. (TIF) [file pone.0126966.s005.tif]

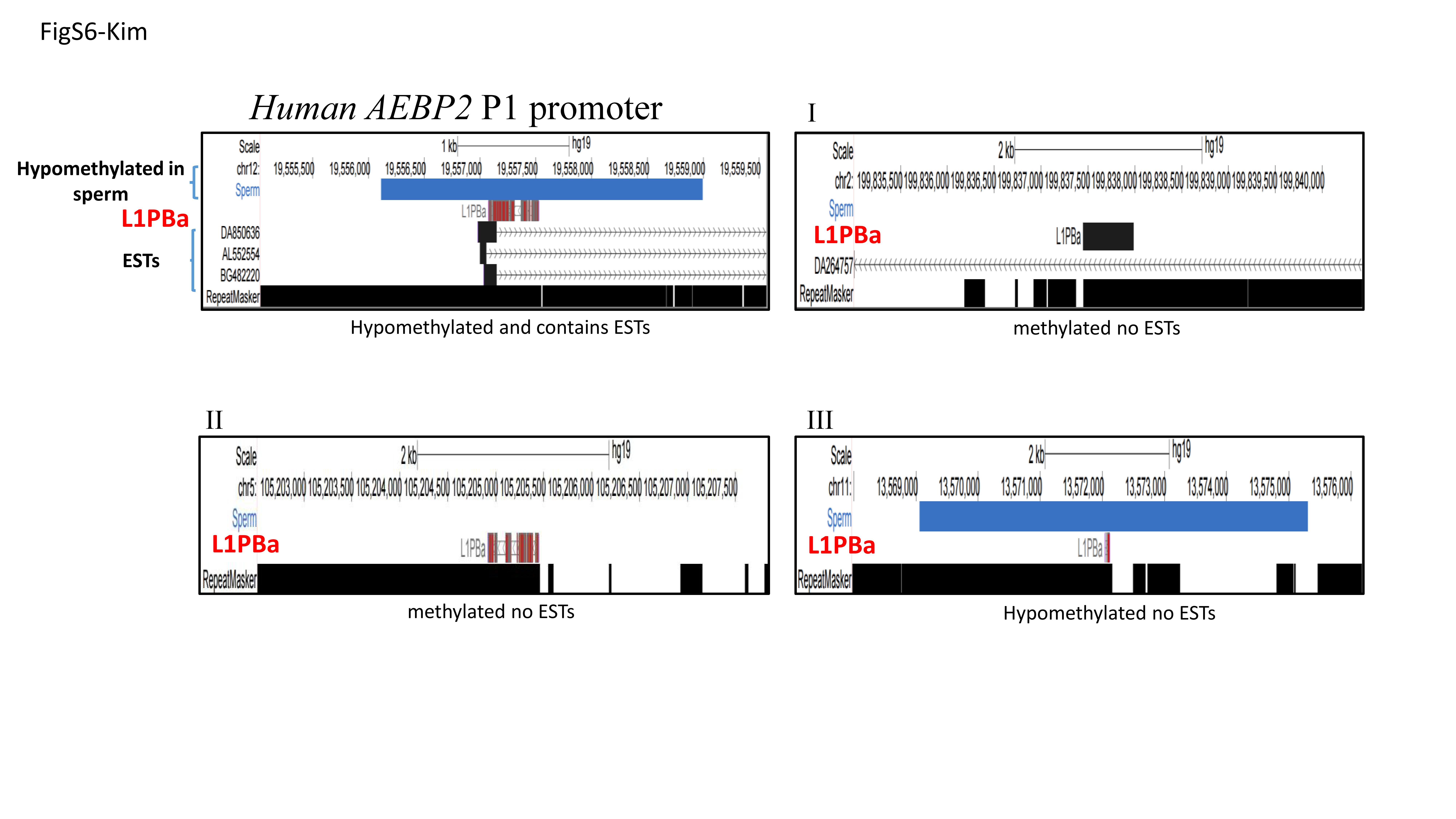

Supplement: S6 Fig — Four snap shots of UCSC genome browsers are from regions marked in S5 Fig, which include regions homologous to L1PBa 5’ UTRs. Hypomethylation of DNA in sperm are marked in blue [17]. Available ESTs are added in the UCSC genome browser tracks. Browsed regions in genome (Hg19): Human AEBP2 P1 chr12:19557074–19557526; (I) chr2:199837446–199837987, (II) chr5:105204915–105205447, (III) chr11:13572036–13572117. (TIF) [file pone.0126966.s006.tif]
